# Communicating the AMFm message: exploring the effect of communication and training interventions on private for-profit provider awareness and knowledge related to a multi-country anti-malarial subsidy intervention

**DOI:** 10.1186/1475-2875-13-46

**Published:** 2014-02-04

**Authors:** Barbara A Willey, Sarah Tougher, Yazoume Ye, Andrea G Mann, Rebecca Thomson, Idrissa A Kourgueni, John H Amuasi, Ruilin Ren, Marilyn Wamukoya, Sergio Torres Rueda, Mark Taylor, Moctar Seydou, Samuel Blay Nguah, Salif Ndiaye, Blessing Mberu, Oumarou Malam, Admirabilis Kalolella, Elizabeth Juma, Boniface Johanes, Charles Festo, Graciela Diap, Didier Diallo, Katia Bruxvoort, Daniel Ansong, Abdinasir Amin, Catherine A Adegoke, Kara Hanson, Fred Arnold, Catherine Goodman

**Affiliations:** 1 Faculty of Epidemiology and Population Health, London School of Hygiene and Tropical Medicine, Keppel Street, London WC1E 7HT, UK; 2 Faculty of Public Health and Policy, London School of Hygiene and Tropical Medicine, Keppel Street, London WC1E 7HT, UK; 3 ICF International, 530 Gaither Rd, Rockville, MD 20850, USA; 4 Malaria & Child Survival Department, Population Services International (PSI), PO Box 43640, Nairobi, Kenya; 5 Ifakara Health Institute, Plot 463, Kiko Avenue Mikocheni, Dar es Salaam, P.O. Box 78 373, Tanzania; 6 Centre International d‘Etudes et de Recherches sur les Populations Africaines (CIERPA), Niamey, Niger; 7 Institut National de la Statistique, 182 Rue de la SIRBA, Niamey BP 3416, Niger; 8 University of Minnesota School of Public Health, Mayo Memorial Building, 420 Delaware Street S.E, Minneapolis, MN 55455, USA; 9 Komfo Anokye Teaching Hospital (KATH), P.O. BOX KS 1934, Kumasi, Ghana; 10 African Population and Health Research Center, APHRC Campus, 2nd Floor, Manga Close, Off Kirawa Road, P.O. Box 10787–00100, Nairobi, Kenya; 11 Department of Public Health, Trnava University, Faculty of Health Sciences and Social Work, Univerzitne namestie 1, Trnava 91701, Slovakia; 12 Centre de Recherche pour le Développement Humain (CRDH), Dakar, Senegal; 13 Kenya Medical Research Institute, Centre for Global Health Research, Kisumu, Kenya; 14 Drugs for Neglected Diseases initiative, 15 Chemin Louis-Dunant, Geneva 1202, Switzerland; 15 Department of Child Health, Kwame Nkrumah University of Science & Technology, School of Medical Sciences, Kumasi, Ghana; 16 Phar-Mark Consultants, Ilorin, Kwara State, Nigeria

## Abstract

**Background:**

The Affordable Medicines Facility - malaria (AMFm), implemented at national scale in eight African countries or territories, subsidized quality-assured artemisinin combination therapy (ACT) and included communication campaigns to support implementation and promote appropriate anti-malarial use. This paper reports private for-profit provider awareness of key features of the AMFm programme, and changes in provider knowledge of appropriate malaria treatment.

**Methods:**

This study had a non-experimental design based on nationally representative surveys of outlets stocking anti-malarials before (2009/10) and after (2011) the AMFm roll-out.

**Results:**

Based on data from over 19,500 outlets, results show that in four of eight settings, where communication campaigns were implemented for 5–9 months, 76%-94% awareness of the AMFm ‘green leaf’ logo, 57%-74% awareness of the ACT subsidy programme, and 52%-80% awareness of the correct recommended retail price (RRP) of subsidized ACT were recorded. However, in the remaining four settings where communication campaigns were implemented for three months or less, levels were substantially lower. In six of eight settings, increases of at least 10 percentage points in private for-profit providers’ knowledge of the correct first-line treatment for uncomplicated malaria were seen; and in three of these the levels of knowledge achieved at endline were over 80%.

**Conclusions:**

The results support the interpretation that, in addition to the availability of subsidized ACT, the intensity of communication campaigns may have contributed to the reported levels of AMFm-related awareness and knowledge among private for-profit providers. Future subsidy programmes for anti-malarials or other treatments should similarly include communication activities.

## Background

Artemisinin-based combination therapy (ACT) is the recommended first-line treatment for uncomplicated *Plasmodium falciparum* infection throughout Africa, however, its use remains far below need and differs between urban and rural areas [1]. The reasons for this include unreliable public sector supply, high prices and limited availability in the private sector, and patient self-treatment with less expensive monotherapies [2]. In 2010, an innovative ACT subsidy programme, the Affordable Medicines Facility - malaria (AMFm), was launched at national scale in Ghana, Kenya, Madagascar, Niger, Nigeria, Uganda, and Tanzania (mainland and Zanzibar) [3]. The programme aimed to improve the availability of quality-assured ACT, and was hosted by the Global Fund to Fight AIDS, Tuberculosis and Malaria (Global Fund).

**Figure 1:**
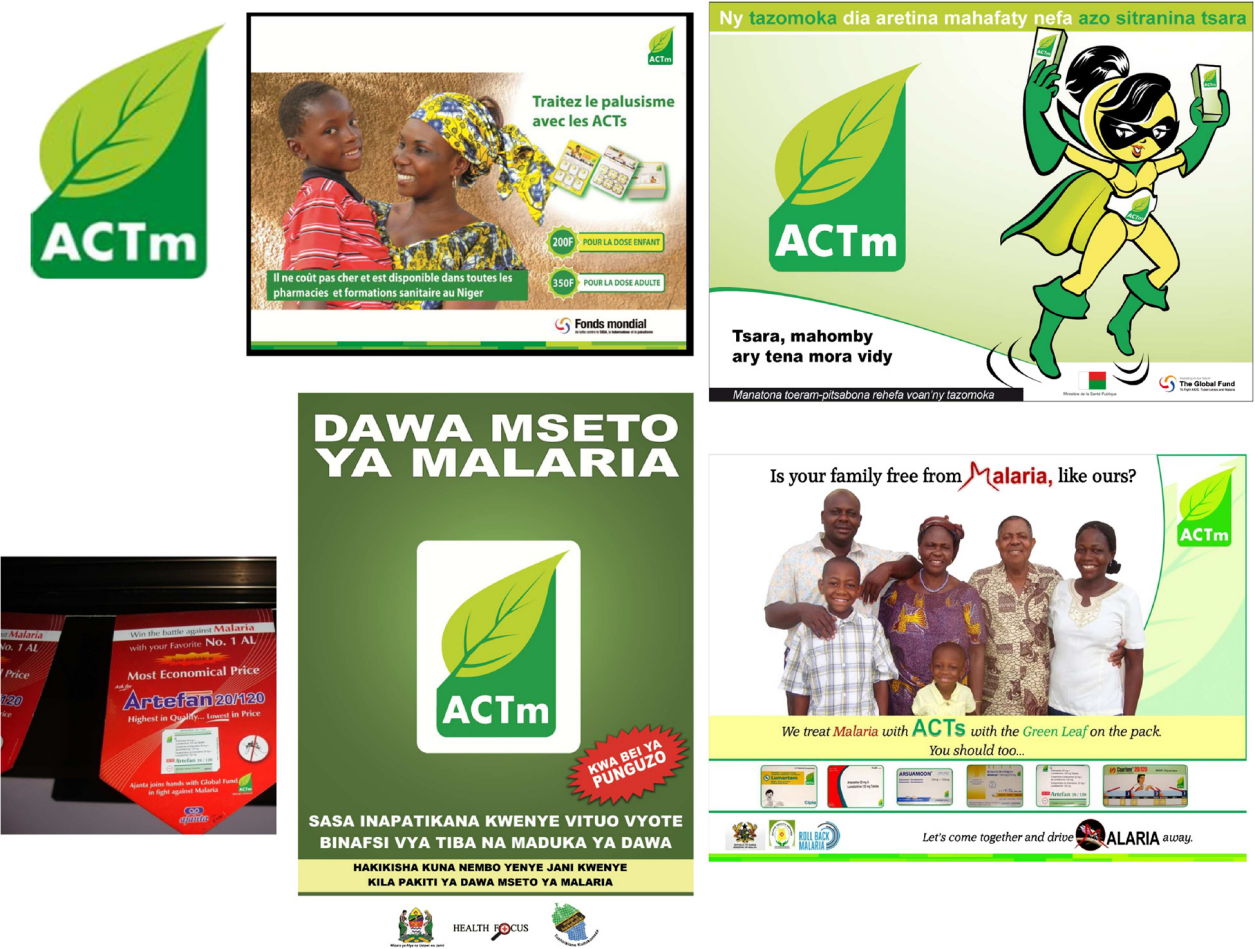
(clockwise from top left) AMFm ‘green leaf’ logo; examples of AMFm communication campaign materials from Niger, Madagascar, Ghana and Kenya; and an example of commercial promotion for co-paid ACT from Ghana.

The AMFm intervention included three elements: (i) price reductions through negotiations with ACT manufacturers; (ii) a buyer subsidy, via a co-payment by the Global Fund to participating manufacturers, for purchases made by eligible public, private, and non-governmental organisation importers; and (iii) interventions to support AMFm implementation and promote appropriate anti-malarial use [4]. All medicines subsidized through AMFm had a ‘green leaf’ logo on their packaging (see logo in Figure 1). Interventions to support AMFm implementation and promote appropriate anti-malarial use included communication campaigns, training of anti-malarial providers, setting of a recommended retail price (RRP), pharmacovigilance and post-marketing surveillance, and regulatory interventions such as enforcement of the ban on oral artemisinin monotherapy sales and changes to the prescription-only status of ACT.

Communication campaigns, including the use of mass media, have been used alone or as components of interventions to address a variety of public health problems in low and middle income countries including HIV prevention, family planning promotion, and the use of insecticide-treated bed nets for malaria control [5]. The effectiveness of such campaigns on behaviour change has been the subject of a number of reviews, and remains a key question for national and international policy makers [6-9]. In the context of AMFm, this is of particular interest given the scale of resources devoted to this component of the intervention ($42.3 million disbursed for supporting interventions overall by the end of 2011) and the relative novelty of the approaches used, such as the AMFm logo and RRPs.

This paper describes the implementation of the AMFm communication and training interventions as part of the overall AMFm intervention package. Private providers of anti-malarials can be considered a key target group for AMFm communication and training, given the geographical penetration of the private for-profit sector, as well as the role of providers in product ordering, retail pricing, point of sale promotion, and patient advice [10-18]. For this reason, this paper focuses on the private for-profit sector, and reports private for-profit anti-malarial provider awareness of key features of the AMFm programme, and changes in provider knowledge of appropriate malaria treatment following AMFm implementation, based on nationally-representative survey data.

## Methods

### Study design and sampling

This study had a non-experimental design based on before-and-after comparisons along with detailed documentation of implementation process and context, as recommended in guidelines for the evaluation of complex interventions [19]. Nationally-representative surveys of outlets stocking anti-malarials were carried out between August and December 2010 prior to arrival of AMFm co-paid ACT, and again between October 2011 and January 2012 [20]. Methods for these surveys were adapted from the ACTwatch project [21].

Outlets were sampled using a stratified cluster sampling approach, with independent samples drawn at baseline and endline. Clusters were administrative units with on average 10,000-15,000 inhabitants. Clusters were selected with probability proportional to population size sampling, with stratification by urban and rural domains. Within a cluster, all outlets with the potential to sell anti-malarials were approached. Eligible outlets included those open at the time of the visit and with anti-malarials in stock on the day of the survey or within the previous three months. A full census of outlets was carried out in Zanzibar due to its small population size.

### Data collection

Data were collected using structured interviews with the most senior staff member present at the time of the survey. Questionnaires were harmonized across settings and created in English and French, with translation into local languages where necessary. Questionnaires were administered by local study staff who had undergone seven days of standardized training. Quality assurance included daily supervision as well as random re-interviewing of 5%-15% of outlets.

Questionnaires covered outlet characteristics as well as provider awareness and knowledge outcomes (see Additional file 1 for questionnaire). Respondents’ awareness of the AMFm ‘green leaf’ logo (located on co-paid ACT packaging and frequently on promotional material), and open-ended questions on the source from which respondents had seen or heard of the logo, and their understanding of the meaning of the logo were included. Additionally, questions were included on awareness of the subsidy programme, and sources from which respondents had seen or heard of the subsidy programme. Respondents were also asked about their awareness of a RRP for co-paid ACT, and what this RRP was. In relation to knowledge, respondents’ knowledge of the first-line recommended anti-malarial for uncomplicated malaria and knowledge of the correct dosing regimen to treat a child under the age of two years (10 kg) with uncomplicated malaria was tested.

Key informant interviews with national level stakeholders and document review were used to collect data on the process of AMFm implementation, including details of supporting interventions, and of other contextual factors that could have affected AMFm related outcomes. Process data were used to devise a number of measures of intensity of implementation of the communication and training supporting interventions [22]. These included months for which co-paid ACT was available, months for which communication campaigns were implemented, per capita disbursements for supporting interventions, and the proportion of private for-profit providers surveyed at endline who reported that they had attended ‘*a training session about anti-malarials with this [AMFm ‘green leaf’ logo] symbol*’.

### Statistical analysis

Data were analysed in Stata v.11. Point estimates were weighted using survey weights and standard errors calculated taking into account the clustered and stratified sampling strategy. Differences in knowledge outcomes between surveys are expressed in terms of the percentage point change and 95% confidence intervals. No confidence intervals are presented for Zanzibar because a complete census of outlets was done. Additional results by urban and rural domains, and stratified by sub-category within the private for-profit sector, are available from the online additional files.

### Ethical approval

Informed oral consent was obtained from all respondents. Ethics approval was obtained from all national ethics committees, and from Institutional Review Boards of ICF International and the London School of Hygiene and Tropical Medicine.

## Results

Table 1 summarizes characteristics of the sample. Response rates were high, and over 9,900 and 9,600 private for-profit outlets were included in baseline and endline surveys, respectively.

**Table 1:** Sample description—number of private for-profit outlets screened and number included in baseline (2010) and endline (2011) outlet surveys

**Country**	**Selected clusters**	Outlets enumerated^*^	**Outlets screened**	Eligible outlets^**^	**Outlets interviewed and stocking anti-malarials at the time of the survey visit**^**†**^
**Ghana**					
*Baseline*	55	1 009	960	942	924
*Endline*	54	752	681	658	646
**Kenya**					
*Baseline*	57	16 356	12 091	2 110	1 457
*Endline*	57	12 512	10 539	1 627	1 378
**Madagascar**					
*Baseline*	38	6 380	6 005	2 064	1 854
*Endline*	46	9 116	8 559	2 081	1 641
**Niger**					
*Baseline*	75	3 104	3 098	1 915	1 548
*Endline*	64	3 102	2 922	1 703	1 337
**Nigeria**					
*Baseline*^‡^	114	5 713	5 171	1 941	1 864
*Endline*	124	8 345	7 804	1 445	1 393
**Tanzania mainland**					
*Baseline*	48	3 042	3 015	612	545
*Endline*	49	3 708	3 635	734	726
**Uganda**					
*Baseline*	39	9 692	9 525	1 733	1 590
*Endline*	44	14 734	14 451	2 453	2 335
**Zanzibar**^‡‡^					
*Baseline*	-	2 100	2 076	177	171
*Endline*	-	4 134	4 057	227	216

*Outlets that were visited and where at a minimum basic descriptive information was collected. **Outlets that had antimalarial drugs in stock on the day of the survey or had stocked them in the past three months. † Outlets where antimalarials were in stock on the day of the survey and the interview was completed. ‡ Nigeria baseline data collection done in 2009. ‡‡ A full census was carried out in Zanzibar.

### Communication and training activities

The planned package of AMFm communication campaigns and promotional activities was broadly similar across settings; components generally included are shown in (Table 2). The AMFm ‘green leaf’ logo together with examples of promotional materials are shown in Figure 1. Promotional messages frequently focused on ACT as recommended anti-malarial treatment, availability of good quality ACT identified by the ‘green leaf’ AMFm logo, and on the RRP (which was typically not printed on ACT packaging, but promoted during communication campaigns).

**Table 2:** Components of the AMFm communication campaigns generally included across all settings


AMFm supporting interventions for communications	
•	National launch;
•	Mass media communication through TV and radio (principally advertisements, with some TV and radio talk shows);
•	Outdoor media (billboards);
•	‘Small media’ (posters);
•	Interpersonal media (community meetings and road shows)
Commercial promotion of co-paid ACT	
•	‘Small’ media provided by importers and wholesalers of co-paid ACT (e.g. branded posters and banners displayed within outlets)

AMFm-related provider training was conducted for providers from all sectors in Ghana, Niger, Nigeria, Uganda and Zanzibar, while private for-profit providers were targeted in Kenya and Tanzania mainland, and the public sector in Madagascar. In some settings, private for-profit importers also carried out training of anti-malarial providers, running courses for their own distributors and sponsoring continuous education meetings for professional bodies (e.g. clinical officers or pharmacists). Private for-profit importers in some of the pilots also produced their own promotional materials (Figure 1).

Countries experienced a range of intervention intensities in the implementation of communication campaigns and training (Table 3). Ghana and Kenya experienced the longest communication campaigns (about nine months), with Tanzania mainland, Zanzibar and Nigeria experiencing three to seven months. By contrast, Niger, Madagascar and Uganda experienced no sustained delivery principally due to delays or suspension of grants. In Ghana and Zanzibar, about 40%-50% of anti-malarial providers interviewed received training. In Kenya, Niger, Uganda and Tanzania mainland this was lower at 12% -18%. In total about 2% of respondents in Madagascar reported receiving some training, although no official AMFm training interventions for private sector providers were reported in this setting. Up until the midpoint of the endline survey, the Global Fund had disbursed almost 42.3 million dollars (USD) for the implementation of supporting interventions, including communication, training, pharmacovigilance, post-marketing surveillance, and regulatory interventions (personal communication Melisse Murray, The Global Fund to Fight AIDS, Tuberculosis and Malaria AMFm secretariat- May 2012). Disbursement per capita ranged from 0.42 USD in Ghana to 0.06 USD in Niger and Madagascar and 0.03 USD in Tanzania mainland.

**Table 3:** Implementation ‘intensity’ of AMFm supporting interventions, including communication and training

**Country**	**Time from arrival of AMFm co-paid ACT to midpoint of endline survey (months)**	Months of implementation of communication campaign preceding the midpoint of the endline survey^*^	**Percentage of private for-profit outlet respondents reporting attending a training session on anti-malarials ‘with this symbol’ (i.e. AMFm logo) at endline**	Disbursement of funding for supporting interventions preceding midpoint of endline survey (USD per capita)^**^
Ghana	15.5	9	50.2	0.42
Kenya	15	9	12.0	0.18
Tanzania mainland	13.5	7	18.1	0.03
Zanzibar	6.5	5	37.5	0.11
Nigeria	9.5	3	13.5	0.10
Niger	7	2	12.8	0.06
Madagascar	14	1	2.2	0.06
Uganda	9.5	0	16.6	0.17^†^

*In some countries there were also some limited communication activities prior to the main roll-out of the communication campaign. For Niger and Madagascar there were some communication campaign activities but these were suspended prior to endline data collection. In Madagascar the radio and TV campaigns ran only from April-May 2011 due to a ban on advertising prescription medication directly to the public except during periods of public health emergencies. ^**^ Figures represent disbursement of Global Fund grants for AMFm supporting interventions including communication campaigns and promotional materials; awareness-building community-based activities; training of anti-malarial providers; pharmacovigilance and post-marketing surveillance; and regulatory interventions – source: Global Fund Secretariat. ^†^For Uganda $0.17 per capita disbursement took place, and although expenditure data was not available disbursement of funds took place less than one month before the midpoint of the endline survey, and no implementation of communication was recorded as taking place in this setting.

**Figure 2:**
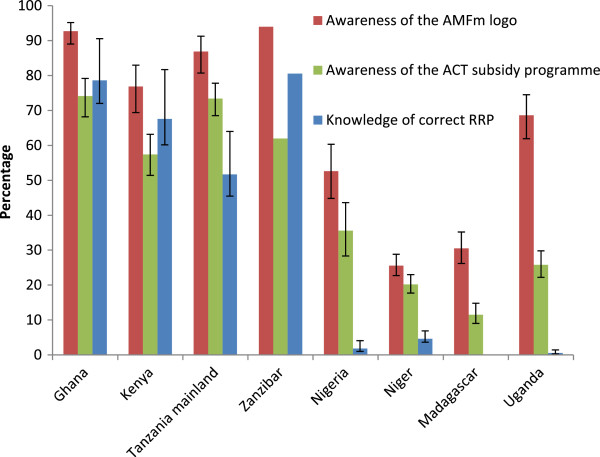
Awareness of the AMFm ‘green leaf’ logo ^*^the ACT subsidy programme, and the correct recommended retail price for co-paid ACT at endline (2011) among respondents from private for-profit outlets with anti-malarials in stock on the day of the survey. ^*^All respondents were shown a visual aid depicting the AMFm logo and were asked whether they have seen the symbol before. Providers are “able to recognise the AMFm logo” if they answer that they have seen the symbol before. Whiskers show 95% confidence intervals. No confidence intervals are shown for Zanzibar as a full census was carried out. Results for Madagascar are not presented as no recommended retail price was set for co-paid ACT in this country. Settings are roughly ordered by intensity of communication campaign and training intervention implementation (see Table 3).

In Figure 2 and Table 4, settings are roughly ordered by intensity of communication campaign and training implementation, with Ghana and Kenya considered to have had the greatest intensity, followed by Tanzania mainland, Zanzibar and Nigeria. Niger, Madagascar and Uganda were considered to have the lowest intensity of implementation.

### Awareness of the AMFm logo, ACT subsidy programme, and correct recommended retail price for co-paid ACT

Overall, awareness of the AMFm logo among anti-malarial providers, measured as whether they remembered having seen the logo before, was 77%-94% in Ghana, Kenya, Tanzania mainland and Zanzibar; somewhat lower in Uganda (69%) and Nigeria (53%); and low in Niger and Madagascar (26% and 31%, respectively) (Figure 2). Recorded awareness in Uganda may have reflected confusion of the AMFm logo with a pre-existing leaf logo used on anti-malarials as part of a separate ACT subsidy project [23]. Awareness among respondents in urban areas was about 7-percentage points higher than among respondents in rural areas in Tanzania mainland and Zanzibar, and between 11–16 percentage points higher than among respondents in rural areas in Ghana, Kenya, Madagascar, and Niger, with no substantial differences in Nigeria or Uganda (Additional file 2). Awareness of the ACT subsidy programme was lower than awareness of the logo, but patterns were broadly similar (Figure 2). In general no substantial differences were seen between urban and rural areas in Madagascar, Nigeria or Tanzania mainland. In Niger, Uganda and Kenya awareness of the ACT subsidy programme was about 6–9 percentage points higher among respondents from urban outlets, and about 12–14 percentage points higher among respondents from urban outlets in Ghana and Zanzibar (Additional file 3).

The most commonly stated source for having seen or heard of the logo was TV/radio in Ghana, Kenya, Madagascar and Zanzibar, with 47%-80% of respondents who had recognised the logo stating this source; and on malaria medicine packaging in Niger, Nigeria, Tanzania mainland and Uganda (50%-58%). The most commonly stated source for having seen or heard of the ACT subsidy programme was TV/radio in all countries with 56%-94% of respondents stating this source. Other sources commonly mentioned were: in training; on malaria medicine packaging; in public health facilities; and on posters/ billboards. Respondents were asked to describe what the AMFm logo meant to them (multiple responses were allowed). The most commonly reported meaning in Ghana, Kenya, Madagascar, Niger and Zanzibar was “effective/quality anti-malarial”. In Nigeria, Tanzania mainland and Uganda, the most common meaning was “ACT.” Other common meanings were “affordable anti-malarial” and “anti-malarial”.

RRPs for co-paid ACT bearing the AMFm logo were set in seven of the eight settings, and ranged between 0.46 and 0.96 USD per adult treatment pack. In Madagascar, there was no RRP, although first-line buyers agreed to maintain a reasonable mark up of 0.07 USD. Awareness of the correct RRP for an adult pack was high among respondents in Ghana and Zanzibar (~80%); and 68% in Kenya and 52% in Tanzania mainland. Respondents in Niger, Nigeria and Uganda had very low awareness of the correct RRP (1%-5%) (Figure 2). Patterns were consistent across urban and rural areas in Nigeria, Tanzania mainland, and Uganda. In Kenya and Niger awareness of the correct RRP among urban respondents was about 9–10 percentage points higher, while in Zanzibar and Ghana awareness of the correct RRP among was 13 and 28 percentage points higher than among rural respondents, respectively (Additional file 4).

### Changes in private for-profit sector provider knowledge of malaria treatment

At baseline knowledge of the first-line treatment for uncomplicated malaria varied across settings, with high knowledge in Ghana, Tanzania mainland, Uganda and Zanzibar, a lower level in Kenya, and low levels in Niger, Nigeria and Madagascar (Table 4). Improvements of over 20 percentage points were recorded in Kenya and Nigeria, and of 7–16 percentage points in Ghana, Madagascar, Niger, Tanzania mainland and Zanzibar, with no change in Uganda. Patterns were consistent across urban and rural areas, with the exception of Tanzania mainland where the increase in knowledge was about 10 percentage points higher among rural respondents surveyed (Additional file 5).

**Table 4:** Knowledge of first-line malaria treatment among respondents from private for-profit outlets with anti-malarials in stock on the day of the survey at baseline (2010) and endline (2011)

**Country**	**Baseline (%)**	**Endline (%)**	**Percentage point change (95% confidence interval)**
Ghana	73.2	83.3	10.1 (3.1-17.3)
Kenya	44.9	66.1	21.2 (11.5-30.8)
Tanzania mainland	85.6	95.7	10.1 (4.0-16.1)
Zanzibar	77.2	92.1	14.9
Nigeria	14.3	51.2	36.9 (28.7-45.2)
Niger	11.1	27.2	16.1 (11.0-21.2)
Madagascar	12.5	19.4	6.9 (0.9-12.7)
Uganda	74.0	74.8	0.8 (−5.6-7.1)

No confidence interval is shown for Zanzibar as a full census was carried out. Settings are roughly ordered by intensity of communication campaign and training intervention implementation (see Table 3).

Generally increases in knowledge of the correct quality-assured ACT dosing regimen for a 2 year-old child were smaller than those seen in respondents’ knowledge of the recommended first-line treatment for uncomplicated malaria among providers surveyed. At endline knowledge of the dosing regimen remained low in many settings, despite increases of between 14–16 percentage points in Ghana and Uganda, and of 30–33 percentage points in Tanzania mainland and Zanzibar. Only in Tanzania mainland were high levels of knowledge recorded at endline (90%) (Table 5). Patterns were consistent across urban and rural areas, except in Uganda where increases in knowledge of the correct quality-assured ACT dosing regimen for children among urban respondents were 12 percentage points larger than those recorded among rural respondents, and Zanzibar where increases in knowledge among rural respondents were about 10 percentage points higher than urban respondents (Additional file 6).

**Table 5:** Knowledge of paediatric (<2 years of age) quality-assured ACT dosing regimen among respondents from private for-profit outlets with quality-assured ACT in stock on the day of the survey at baseline (2010) and endline (2011)

**Country**	**Baseline (%)**	**Endline (%)**	**Percentage point change (95% confidence interval)**
Ghana	31.4	47.8	16.4 (8.7-24.0)
Kenya	67.4	60.6	−6.8 (−17.0-3.5)
Tanzania mainland	60.0	89.5	29.5 (11.7-47.1)
Zanzibar	15.4	48.7	33.3
Nigeria^*^	-	53.7	-
Niger	64.1	43.1	−21.0 (−34.3-7.8)
Madagascar^*^	-	41.6	-
Uganda	64.1	78.5	14.4 (3.3-25.4)

Correct knowledge of paediatric quality-assured ACT dosing regimen was measured as respondents that correctly stated the number of tablets that should be taken at a time, the number of times the medicine should be taken per day, and the duration of the dose in number of days for child under 2 years (10 kg) for a specific product which they selected from the quality-assured ACT that they stocked.

Nigeria baseline data collection was conducted in 2009. No confidence interval is shown for Zanzibar as a full census was carried out. Settings are roughly ordered by intensity of communication campaign and training intervention implementation (see Table 3).

^*^These data are not available for Madagascar and Nigeria at baseline, as they were not collected in the ACTwatch survey.

## Conclusions

Although AMFm implementation was designed to be broadly similar across settings, the communication and training components varied considerably across the eight pilots. Ghana and Kenya had the greatest implementation intensity, followed by Tanzania mainland, Zanzibar and Nigeria, with Niger, Madagascar and Uganda having the lowest intensity.

Our results generally support the interpretation that, in addition to the availability of co-paid ACT, the intensity of communication campaigns, and particularly the mass media elements delivered through TV and radio, contributed to AMFm-related awareness and knowledge among private for-profit providers. For example, in settings where communication campaigns were implemented for five to nine months, awareness was 76%-94% for the AMFm logo, 57%-74% for the ACT subsidy programme, and 52%-80% for the correct RRP. Where communication campaigns were implemented for three months or less however, awareness was 26%-69% for the logo, 12%-36% for the ACT subsidy programme, and <5% for the RRP.

Communication campaigns also appear to have contributed to improvements in provider knowledge of the first-line anti-malarial. In six of eight settings, increases of at least 10 percentage points in private for-profit providers’ knowledge of the first-line anti-malarial were seen; with endline knowledge particularly high in Kenya (66%), Ghana (83%) and Tanzania (mainland and Zanzibar) (over 90%). In contrast, much smaller improvements were seen in Madagascar and Uganda. By comparison, in non-AMFm settings private for-profit provider knowledge of the first-line anti-malarial ranged from 42%-48% across Benin, the Democratic Republic of Congo and Zambia [24-26].

AMFm-related training also appeared to be linked to knowledge and awareness outcomes, with the two settings with the highest training coverage (Ghana and Zanzibar) performing best at endline on knowledge of the logo and RRP, and being in the top three settings for knowledge of the first-line anti-malarial. However, high levels of knowledge in some settings with relatively low training coverage indicate that providers also obtain information through other sources, such as communications targeted at the general public, or through wholesale suppliers.

Given the study design, caution is merited in making strong causal inferences about the impact of AMFm supporting interventions. The evaluation was ecological in design, with exposure estimated at the national level using proxy measures of duration of implementation. Moreover, the nationwide nature of AMFm implementation meant that it was not possible to include comparison areas. While AMFm-specific awareness is clearly related to some aspect of the AMFm programme, changes in knowledge of the first-line anti-malarial and quality-assured ACT dosing regimen for children may have been affected by secular trends or other concurrent malaria communication campaigns, such as the large scale USAID-funded Communication and Malaria Initiative in Tanzania (COMMIT) programme in mainland Tanzania [27].

Furthermore, these results report awareness among private for-profit providers only. Awareness among consumers has been reported to be substantially lower in several settings, though results are only available from settings with medium to low AMFm implementation intensity. Findings from nationally representative household surveys in Nigeria, Madagascar and Uganda in 2012 indicate that 13%-40% of caregivers of children aged less than five years recognised the AMFm logo, while 9%-18% were aware of the initiative to reduce the price of ACT. Data from Nigeria and Uganda (where an RRP was used) show that only 0.1% of those surveyed were aware of the correct RRP for co-paid ACT [28,29].

There also appears to be a relationship between private for-profit sector providers’ AMFm-related awareness and key AMFm outcomes. Countries with the strongest performance in AMFm awareness (Ghana, Kenya, Tanzania and Zanzibar) generally reported larger increases in availability and market share of quality-assured ACT in the private for-profit sector; larger falls in their price compared to smaller changes seen in Nigeria and Uganda on the whole; and minimal changes in Niger and Madagascar [20]. In contrast, the relationship between improvements in knowledge of the first-line anti-malarial and AMFm outcomes were inconsistent. It is thus possible that AMFm-related provider awareness may be an important step along a causal pathway, linking implementation to provider behaviour change, though in practice it is challenging to separate the effect of communications from the reduced price of the co-paid ACT. Evaluations of mass media campaigns for other health issues in low and middle-income countries have shown links to behaviour change of community members [6,30,31]. However, other studies have highlighted the frequent presence of a knowledge-action gap among health care providers [14].

This study has demonstrated that in settings with strong implementation of communication campaigns, and in some cases provider training, there was high AMFm-related awareness among private for-profit providers within a short period of AMFm roll out. Substantial improvements in provider’s knowledge of the first-line drug were also seen. These results suggest an important role for supporting interventions, including communication campaigns, in subsidy programmes for public health commodities. However, to optimize investment in this area more evidence is required on the relative effectiveness of different supporting interventions on providers, and on strategies to enhance consumer awareness.

## Competing interests

All authors declare that they have no conflicts of interest.

## Authors’ contributions

The overall design of the Independent Evaluation of AMFm was conceived by FA, KH, YY, CG, and ST. The design of the outlet surveys drew heavily on data collection and analysis methods developed by the ACTwatch group. Adaptation of outlet survey data collection methods for the Independent Evaluation was led by ST, CG, KH, YY, FA, and RR, and development of procedures for outlet survey data analysis was led by AM, ST, BW, CG, KH, and YY with significant contributions from HG and SP of the ACTwatch group and with inputs from many other authors. Outlet survey data collection and analysis was led by the ACTwatch group in Kenya (MW, BM, MT, KOC, IE, SP, JN, TS, HG), Madagascar (JR, JR, JN, SP), Nigeria (EA, JA, KOC, IE, HG, JN), Uganda (PB, HK, JN, IE, SP, KOC, HG), and Zanzibar (DM, JN, HG); by the Ifakara Health Institute (RT, BJ, AK, MT, CF, KB) in Tanzania mainland; by the Drugs for Neglected Diseases initiative (DNDi)/Research and Development Unit, Komfo Anokye Teaching Hospital (JA, GD, SBN, DA) in Ghana; by the Centre de Recherche pour le Développement Humain/Centre International d’Etudes et de Recherches sur les Populations Africaines (SN, IAK, MS, OM) in Niger; and the African Population and Health Research Centre (MW, BM) at endline in Kenya. Analysis of changes over time in outlet survey indicators was conducted by AM, ST, BW, and RR. The process and context study component was designed by ST, KH, CG, FA, and YY, with data collection and analysis undertaken by EJ, AA, STR, DD, CA, CG, ST, and YY. FA was the project director for the Independent Evaluation. KH led the Independent Evaluation team at the London School of Hygiene and Tropical Medicine. The first draft of the paper was written by BW, with inputs from many authors. All authors have read and approved the final manuscript.

## Supplementary Material

Additional file 1 AMFm questionnaire. Click here for file

Additional file 2 **Provider recognition of AMFm logo at endline (2011).** Provider recognition of AMFm logo (i.e. Providers able to recognise the AMFm logo (n) as a percentage of the number of outlets with anti-malarials in stock at the time of the survey visit (N )) at endline (2011), by anti-malarial outlet type category and urban and rural location. Note: All respondents were shown a visual aid depicting the AMFm logo and were asked whether they had seen the symbol before. Providers were “able to recognise the AMFm logo” if they answered that they had seen the symbol before. CI = Confidence interval; No confidence intervals are shown for Zanzibar as a full census was carried out. Click here for file

Additional file 3 **Provider knowledge of the AMFm programme at endline (2011).** Provider knowledge of the AMFm programme (i.e. Providers who have heard of “a programme that reduces the prices of anti-malarial medicines known as ACT” (n) as a percentage of outlets with anti-malarials in stock at the time of the survey visit (N)) at endline (2011), by anti-malarial outlet type category and urban and rural location. Footnote: CI = Confidence interval; No confidence intervals are shown for Zanzibar as a full census was carried out. Click here for file

Additional file 4 **Providers stating the correct recommended retail price (RRP) for anti-malarials with the AMFm logo at endline (2011).** Providers stating the correct recommended retail price (RRP) for anti-malarials with the AMFm logo at endline (2011) (i.e. Providers stating the correct RRP for anti-malarials with the AMFm logo (n) as a percentage of outlets with anti-malarials in stock at the time of the survey visit (N)) at endline (2011), by anti-malarial outlet type category and urban and rural location.Note: No data are shown for Madagascar as an RRP was not set for co-paid ACTs in this country, CI = Confidence interval; No confidence intervals are shown for Zanzibar as a full census was carried out. Click here for file

Additional file 5 **Provider knowledge of first-line anti-malarial treatment at baseline (2010) and endline (2011).** Provider knowledge of first-line anti-malarial treatment at baseline (2010) and endline (2011) (i.e. Percentage of providers able to correctly identify the anti-malarial for first-line treatment (n) among outlets with anti-malarials in stock at the time of the survey visit (N)) baseline (2010) and at endline (2011), by anti-malarial outlet type category and urban and rural location.Note: Nigeria baseline data collection was conducted in 2009. CI = Confidence interval; No confidence intervals are shown for Zanzibar as a full census was carried out. Click here for file

Additional file 6 **Provider knowledge of dosing regimen for quality-assured ACT (QAACT) for a child, at baseline (2010) and endline (2011).** Provider knowledge of dosing regimen for quality-assured ACT (QAACT) for a child, at baseline (2010) and endline (2011) (i.e. Percentage of providers able to describe correctly the dosing regimen for quality-assured ACT for a child under 2 years of age (<10 kg) (n) among outlets with QAACT in stock at the time of the survey visit (N)) baseline (2010) and at endline (2011), by anti-malarial outlet type category and urban and rural location. Note: “describe correctly” implies that the respondent correctly stated the number of tablets that should be taken at a time, the number of times the medicine should be taken per day and the duration of the dose in number of days for child under 2 years (10kg) for a specific product which they selected from the quality-assured ACTs that they stocked. These data are not available for Madagascar and Nigeria at baseline, as they were not collected in the ACTwatch survey. Nigeria baseline data collection was conducted in 2009. CI = Confidence interval; No confidence intervals are shown for Zanzibar as a full census was carried out. Click here for file

## Collaborators

The ACTwatch Group (ncharman@psi.org): ACTwatch Central—Population Services International, Nairobi, Kenya (Gatakaa H, Poyer S, Njogu J, Evance I, Littrell M, Chavasse D, O’Connell K [Principal Investigator], and Shewchuk T); ACTwatch Central—London School of Hygiene and Tropical Medicine, London, UK (Palafox B); ACTwatch National Level—Kenya: Population Services International (Toda M); Madagascar: Population Services International/Madagascar (Raharinjatovo J, Rahariniaina J); Nigeria: Society for Family Health (Anyanti J, Arogundade E); Tanzania: Population Services International (Michael D); and Uganda: PACE (Buyungo P, Kuala H).
