# Supplementary material for: Communicating the AMFm message: exploring the effect of communication and training interventions on private for-profit provider awareness and knowledge related to a multi-country anti-malarial subsidy intervention
Source: Malar J. 2014 Feb 4;13:46. doi: 10.1186/1475-2875-13-46 (PMC3924415; doi:10.1186/1475-2875-13-46)
Supplement: Additional file 1 — AMFm questionnaire. [file 1475-2875-13-46-S1.docx]

Figure web 1: AMFm questionnaire

Provider Awareness of AMFm logo:

| P10. Have you seen or heard of this symbol before? ***Show prompt card with AMFm logo***  1 = Yes  0 = No ***go to P13***  8 = Don’t know ***go to P13*** | [___] |
| --- | --- |
| P11. Where have you seen or heard of this symbol before? ***Do not read list***. ***Multiple responses allowed. Repeat prompt “anywhere else” until no more suggestions are provided***  1 = response mentioned  0 = response not mentioned   1. On malaria medicine packaging 2. On medicine packaging 3. On posters 4. On billboards 5. On TV/radio 6. On a prescription 7. In newspapers/magazines 8. In pharmacies/ drug shops 9. In private clinics 10. In public health facilities 11. In training 12. From a supplier 13. From a public event 14. From a local leader 15. From a friend/family member 16. On the internet 17. Don’t Know 18. Other ***(specify)***   [_______________________________________________]  [_______________________________________________]  [_______________________________________________] | [___]  [___]  [___]  [___]  [___]  [___]  [___]  [___]  [___]  [___]  [___]  [___]  [___]  [___]  [___]  [___]  [___] |
| P12. What does this symbol mean to you? ***Do not read list***. ***Multiple responses allowed. Repeat prompt “anything else” until no more suggestions are provided***  1 = response mentioned  0 = response not mentioned     1. Effective/quality antimalarial 2. Affordable antimalarial 3. An antimalarial in high demand 4. Effective/quality medicine 5. Affordable medicine 6. A medicine in high demand 7. It means nothing 8. I don’t know what it means 9. Other ***(specify)***   [_______________________________________________]  [_______________________________________________]  [_______________________________________________] | [___]  [___]  [___]  [___]  [___]  [___]  [___]  [___]  [___] |

Provider Awareness of AMFm subsidy programme:

| N1 Have you heard of the programme that reduces the prices of antimalarial medicines known as ACTs?  1 = Yes  0 = No ***Go to N3***  8 **=** Don’t know ***Go to N3*** | [___] |
| --- | --- |

| N2. How did you hear about the program? ***Do not read list***. ***Multiple responses allowed. Repeat prompt “anything else” until no more suggestions are provided***  1 = response mentioned  0 = response not mentioned   1. On malaria medicine packaging 2. On medicine packaging 3. On posters 4. On billboards 5. On TV/radio 6. On a prescription 7. In newspapers/magazines 8. In pharmacies/ drug shops 9. In private clinics 10. In public health facilities 11. In training 12. From a supplier (including medical representative) 13. From a public event 14. From a local leader 15. From a friend/family member 16. SMS messages 17. On the internet 18. Don’t Know 19. Other ***(specify)***   [________________________________________________]  [________________________________________________]  [________________________________________________] | [___]  [___]  [___]  [___]  [___]  [___]  [___]  [___]  [___]  [___]  [___]  [___]  [___]  [___]  [___]  [___]  [___]  [___] |
| --- | --- |

Recommended Retail Price:

| N3. Are there maximum/ recommended retail prices for antimalarials with this symbol? ***Show prompt card with AMFm logo***  1= Yes  0 = No ***go to N5***  8 = Don’t know ***go to N5*** | [___] |
| --- | --- |
| N4. What is the maximum/ recommended retail price for an adult dose?  9998 = Don’t know | [__\|__\|__\|__] LCU |

Provider Knowledge of recommended treatment for uncomplicated malaria:

| P15. Please name the first line medicine recommended by the government to treat uncomplicated malaria fever. ***Do not read list. Only one response allowed.***  01 = Insert name of government’s first line treatment(s)  02 = Amodiaquine  03 = Artemether  04 = Artemether Lumefantrine  05 = Artemisinin  06 = Artesunate  07 = Artesunate Amodiaquine  08 = Chloroquine  09 = Dihydroartemisinin Piperaquine  10 = Halofantrine  11 = Mefloquine  12 = Quinine  13 = Sulfadoxine Pyrimethamine  96 = Other ***(specify)***: [_______________________________]  98 = Don’t know |
| --- |

Provider Knowledge of pediatric dosing regimen for uncomplicated malaria:

| P22. Please explain the dosing regimen of any one of these products ***(show all gathered QAACT antimalarials)*** for a child under 2 (10kg). ***Read the following 3 questions to the provider***   1. How many tablets should they take at a time? [___\|___].[___\|___] 2. How many times per day? [___\|___] 3. Over how many days? [___\|___]   95 = Not applicable, I would not give/sell any of these products to a child  98 = Don’t know  ***Record the following information from the package of the drug selected by the provider***   \|  \| **Generic name** \| **Strength** \| **Brand Name** \| **Manufacturer** \| \| --- \| --- \| --- \| --- \| --- \| \| [__\|__] \| **________________**  **________________** \| [__\|__\|__].[__]mg  [__\|__\|__].[__]mg  [__\|__\|__].[__]mg \|  \|  \| \| [__\|__] \| \| [__\|__] \| \|  \| Do not write here  [___\|___] \| |
| --- | --- | --- | --- | --- | --- | --- | --- | --- | --- | --- | --- | --- | --- | --- |
