# Supplementary material for: Communicating the AMFm message: exploring the effect of communication and training interventions on private for-profit provider awareness and knowledge related to a multi-country anti-malarial subsidy intervention
Source: Malar J. 2014 Feb 4;13:46. doi: 10.1186/1475-2875-13-46 (PMC3924415; doi:10.1186/1475-2875-13-46)
Supplement: Additional file 2 — Provider recognition of AMFm logo at endline (2011). Provider recognition of AMFm logo (i.e. Providers able to recognise the AMFm logo (n) as a percentage of the number of outlets with anti-malarials in stock at the time of the survey visit (N )) at endline (2011), by anti-malarial outlet type category and urban and rural location. Note: All respondents were shown a visual aid depicting the AMFm logo and were asked whether they had seen the symbol before. Providers were “able to recognise the AMFm logo” if they answered that they had seen the symbol before. CI = Confidence interval; No confidence intervals are shown for Zanzibar as a full census was carried out. [file 1475-2875-13-46-S2.docx]

| **Table web 1: Provider recognition of AMFm logo at endline (2011)** | | | | | | |  |
| --- | --- | --- | --- | --- | --- | --- | --- |
| Providers able to recognise the AMFm logo (n) as a percentage of the number of outlets with antimalarials in stock at the time of the survey visit (N), by urban-rural location and type of outlet, according to country | | | | | | |  |
| **Country/Type of outlet** | **ENDLINE** | | | | | |  |
|  | Urban | | Rural | | Total | | |
|  | % (95% CI) | N | % (95% CI) | N | % (95% CI) | N | |
| **Ghana** |  |  |  |  |  |  | |
| Private for-profit outlet |  |  |  |  |  |  | |
| *Health facility/pharmacy* | 100.0 | 270 | 100.0 | 26 | 100.0 | 296 | |
| *Drug store* | 96.6 (93.3-98.3) | 201 | 83.0 (74.1-89.3) | 140 | 91.3 (87.0-94.2) | 341 | |
| *General retailer/itinerant* | 64.5 (20.5-92.8) | 3 | 66.3 (36.4-87.2) | 3 | 65.4 (37.3-85.7) | 6 | |
| *Total* | 97.2 (94.7-98.5) | 474 | 84.0 (75.2-90.1) | 169 | 92.7 (89.0-95.2) | 643 | |
| **Kenya** |  |  |  |  |  |  | |
| Private for-profit outlet |  |  |  |  |  |  | |
| *Health facility/pharmacy* | 92.2 (88.9-94.6) | 408 | 90.0 (83.0-94.4) | 113 | 90.9 (86.6-93.9) | 521 | |
| *Drug store* | 97.9 (95.1-99.1) | 329 | 95.6 (90.0-98.2) | 145 | 96.5 (93.4-98.2) | 474 | |
| *General retailer/itinerant* | 45.3 (36.9-54.0) | 155 | 50.0 (38.4-61.5) | 222 | 49.1 (39.5-58.8) | 377 | |
| *Total* | 84.5 (78.0-89.4) | 892 | 73.4 (62.8-81.9) | 480 | 76.9 (69.4-83.0) | 1,372 | |
| **Madagascar** |  |  |  |  |  |  | |
| Private for-profit outlet |  |  |  |  |  |  | |
| *Health facility/pharmacy* | 96.4 (93.5-98.1) | 105 | 50.5 (24.3-76.4) | 12 | 75.6 (54.9-88.8) | 117 | |
| *Drug store* | 96.8 (89.4-99.1) | 28 | 70.5 (62.1-77.8) | 347 | 73.3 (65.4-80.0) | 375 | |
| *General retailer/itinerant* | 30.1 (24.0-36.9) | 743 | 23.6 (18.4-29.9) | 405 | 24.5 (19.9-29.9) | 1,148 | |
| *Total* | 43.7 (38.4-49.1) | 876 | 28.2 (23.4-33.6) | 764 | 30.5 (26.2-35.2) | 1,640 | |
| **Niger** |  |  |  |  |  |  | |
| Private for-profit outlet |  |  |  |  |  |  | |
| *Health facility/pharmacy* | 86.8 (79.3-91.8) | 95 | 100.0 | 4 | 87.5 (80.4-92.3) | 99 | |
| *Drug store* | 96.5 (87.3-99.1) | 15 | 30.7 (5.6-77.0) | 3 | 59.9 (30.0-83.9) | 18 | |
| *General retailer/itinerant* | 33.3 (27.9-39.1) | 710 | 20.9 (17.3-25.0) | 510 | 24.3 (21.3-27.6) | 1,220 | |
| *Total* | 36.9 (32.1-42.1) | 820 | 21.1 (17.6-25.1) | 517 | 25.6 (22.7-28.8) | 1,337 | |
| **Nigeria** |  |  |  |  |  |  | |
| Private for-profit outlet |  |  |  |  |  |  | |
| *Health facility/pharmacy* | 77.7 (66.1-86.1) | 99 | 59.6 (44.6-72.9) | 32 | 71.1 (61.5-79.1) | 131 | |
| *Drug store* | 52.0 (38.7-65.0) | 807 | 52.0 (43.0-60.9) | 362 | 52.0 (43.0-60.9) | 1,169 | |
| *General retailer/itinerant* | 35.3 (17.8-58.0) | 74 | 15.4 (5.4-36.6) | 19 | 30.3 (16.6-48.8) | 93 | |
| *Total* | 53.3 (41.9-64.5) | 980 | 51.3 (42.8-59.8) | 413 | 52.6 (44.8-60.3) | 1,393 | |
| **Tanzania - mainland** |  |  |  |  |  |  | |
| Private for-profit outlet |  |  |  |  |  |  | |
| *Health facility/pharmacy* | 91.7 (75.9-97.5) | 321 | 44.7 (12.1-82.7) | 16 | 79.5 (55.5-92.4) | 337 | |
| *Drug store* | 90.5 (85.0-94.2) | 259 | 87.3 (75.0-94.0) | 113 | 88.7 (81.8-93.2) | 372 | |
| *General retailer/itinerant* | 100.0 | 5 | 70.0 (31.0-92.4) | 12 | 72.7 (37.4-92.2) | 17 | |
| *Total* | 90.8 (86.5-93.9) | 585 | 83.7 (73.0-90.7) | 141 | 86.9 (80.7-91.3) | 726 | |
| **Uganda** |  |  |  |  |  |  | |
| Private for-profit outlet |  |  |  |  |  |  | |
| *Health facility/pharmacy* | 74.4 (69.6-78.6) | 814 | 76.2 (70.7-81.0) | 385 | 75.4 (72..0-78.5) | 1,199 | |
| *Drug store* | 66.5 (52.8-78.0) | 435 | 67.8 (57.8-76.4) | 673 | 67.6 (58.9-75.2) | 1,108 | |
| *General retailer/itinerant* | 0 | 3 | 3.5 (0.4-25.4) | 14 | 3.4 (0.4-24.3) | 17 | |
| *Total* | 70.7 (61.2-78.8) | 1,252 | 67.9 (59.8-75.0) | 1,072 | 68.6 (61.9-74.5) | 2,324 | |
| **Zanzibar** |  |  |  |  |  |  | |
| Private for-profit outlet |  |  |  |  |  |  | |
| *Health facility/pharmacy* | 98.8 | 82 | 93.8 | 16 | 98.0 | 98 | |
| *Drug store* | 94.3 | 88 | 87.5 | 24 | 92.9 | 112 | |
| *General retailer/itinerant* | 33.3 | 3 | 66.7 | 3 | 50.0 | 6 | |
| *Total* | 95.4 | 173 | 88.4 | 43 | 94.0 | 216 | |
| Note: All respondents were shown a visual aid depicting the AMFm logo and were asked whether they had seen the symbol before. Providers were “able to recognise the AMFm logo” if they answered that they had seen the symbol before. CI = Confidence interval; No confidence intervals are shown for Zanzibar as a full census was carried out. | | | | | | | |
