# Supplementary material for: Communicating the AMFm message: exploring the effect of communication and training interventions on private for-profit provider awareness and knowledge related to a multi-country anti-malarial subsidy intervention
Source: Malar J. 2014 Feb 4;13:46. doi: 10.1186/1475-2875-13-46 (PMC3924415; doi:10.1186/1475-2875-13-46)
Supplement: Additional file 3 — Provider knowledge of the AMFm programme at endline (2011). Provider knowledge of the AMFm programme (i.e. Providers who have heard of “a programme that reduces the prices of anti-malarial medicines known as ACT” (n) as a percentage of outlets with anti-malarials in stock at the time of the survey visit (N)) at endline (2011), by anti-malarial outlet type category and urban and rural location. Footnote: CI = Confidence interval; No confidence intervals are shown for Zanzibar as a full census was carried out. [file 1475-2875-13-46-S3.docx]

| **Table web 2: Provider knowledge of the AMFm programme at endline (2011)** | | | | | | | |
| --- | --- | --- | --- | --- | --- | --- | --- |
| Providers who have heard of “a programme that reduces the prices of antimalarial medicines known as ACTs” (n) as a percentage of outlets with antimalarials in stock at the time of the survey visit (N), by urban-rural location and type of outlet, according to country | | | | | | | |
|  | Urban | | Rural | | Total | |  |
| **Country/Type of outlet** | % (95% CI) | N | % (95% CI) | N | % (95% CI) | N |  |
| **Ghana** |  |  |  |  |  |  |  |
| Private for-profit outlet |  |  |  |  |  |  |  |
| *Health facility/pharmacy* | 84.5 (78.7-89.0) | 269 | 89.2 (69.3-96.8) | 26 | 85.1 (79.8-89.2) | 295 |  |
| *Drug store* | 75.7 (68.7-81.5) | 202 | 64.7 (52.6-75.1) | 140 | 71.4 (64.9-77.0) | 342 |  |
| *General retailer/itinerant* | 100.0 | 3 | 32.7 (3.8-85.7) | 3 | 66.2 (22.2-93.1) | 6 |  |
| *Total* | 78.3 (72.5-83.1) | 474 | 65.9 (53.7-76.3) | 169 | 74.1 (68.2-79.2) | 643 |  |
| **Kenya** |  |  |  |  |  |  |  |
| Private for-profit outlet |  |  |  |  |  |  |  |
| *Health facility/pharmacy* | 72.3 (62.8-80.1) | 406 | 65.9 (49.8-79.0) | 112 | 68.4 (58.2-77.0) | 518 |  |
| *Drug store* | 70.9 (65.2-76.0) | 328 | 67.3 (57.0-76.2) | 145 | 68.7 (62.1-74.7) | 473 |  |
| *General retailer/itinerant* | 36.5 (24.7-50.3) | 155 | 40.3 (30.7-50.7) | 223 | 39.7 (31.4-48.6) | 378 |  |
| *Total* | 63.7 (56.4-70.4) | 889 | 54.6 (46.6-62.4) | 480 | 57.4 (51.4-63.2) | 1,369 |  |
| **Madagascar** |  |  |  |  |  |  |  |
| Private for-profit outlet |  |  |  |  |  |  |  |
| *Health facility/pharmacy* | 38.3 (30.9-46.3) | 105 | 25.8 (10.0-52.2) | 12 | 32.7 (22.4-44.9) | 117 |  |
| *Drug store* | 14.5 (7.6-25.7) | 28 | 16.2 (12.0-21.4) | 347 | 16.0 (12.2-20.7) | 375 |  |
| *General retailer/itinerant* | 9.3 (7.4-11.7) | 742 | 10.3 (7.1-14.7) | 404 | 10.1 (7.3-13.9) | 1,146 |  |
| *Total* | 13.9 (11.6-16.6) | 875 | 11.1 (8.2-15.0) | 763 | 11.5 (9.0-14.8) | 1,638 |  |
| **Niger** |  |  |  |  |  |  |  |
| Private for-profit outlet |  |  |  |  |  |  |  |
| *Health facility/pharmacy* | 58.9 (49.2-67.9) | 95 | 91.6 (61.9-98.6) | 4 | 60.7 (51.0-69.7) | 99 |  |
| *Drug store* | 66.1 (44.6-82.5) | 15 | 0 | 3 | 29.3 (13.9-51.6) | 18 |  |
| *General retailer/itinerant* | 21.9 (18.7-25.5) | 703 | 18.5 (15.2-22.3) | 503 | 19.5 (16.9-22.3) | 1206 |  |
| *Total* | 24.5 (21.5-27.8) | 813 | 18.5 (15.2-22.3) | 510 | 20.2 (17.7-23.0) | 1323 |  |
| **Nigeria** |  |  |  |  |  |  |  |
| Private for-profit outlet |  |  |  |  |  |  |  |
| *Health facility/pharmacy* | 54.3 (41.6-66.4) | 94 | 55.9 (32.2-77.3) | 31 | 54.9 (42.7-66.5) | 125 |  |
| *Drug store* | 35.1 (24.3-47.7) | 793 | 33.6 (23.1-45.9) | 358 | 34.5 (26.5-43.5) | 1,151 |  |
| *General retailer/itinerant* | 19.7 (14.8-25.7) | 71 | 19.1 (6.8-43.3) | 19 | 19.5 (14.0-26.5) | 90 |  |
| *Total* | 35.8 (26.4-46.5) | 958 | 35.1 (24.5-47.4) | 408 | 35.6 (28.3-43.6) | 1,366 |  |
| **Tanzania - mainland** |  |  |  |  |  |  |  |
| Private for-profit outlet |  |  |  |  |  |  |  |
| *Health facility/pharmacy* | 83.8 (65.5-93.4) | 313 | 84.8 (43.6-97.6) | 16 | 84.1 (68.2-92.9) | 329 |  |
| *Drug store* | 72.7 (64.6-79.5) | 256 | 74.0 (66.0-80.7) | 113 | 73.4 (67.9-78.4) | 369 |  |
| *General retailer/itinerant* | 46.6 (10.9-86.2) | 4 | 59.5 (48.7-69.4) | 12 | 58.5 (47.3-68.9) | 16 |  |
| *Total* | 74.0 (67.5-79.6) | 573 | 72.9 (65.7-79.1) | 141 | 73.4 (68.5-77.8) | 714 |  |
| **Uganda** |  |  |  |  |  |  |  |
| Private for-profit outlet |  |  |  |  |  |  |  |
| *Health facility/pharmacy* | 36.4 (34.7-38.1) | 805 | 32.2 (27.1-37.7) | 384 | 34.0 (30.9-37.3) | 1,189 |  |
| *Drug store* | 25.6 (20.1-32.0) | 433 | 22.5 (18.5-27.1) | 674 | 23.0 (19.4-27.0) | 1,107 |  |
| *General retailer/itinerant* | 0 | 4 | 3.5 (0.4-25.4) | 14 | 3.3 (0.4-23.8) | 18 |  |
| *Total* | 31.5 (27.5-35.8) | 1,242 | 24.1 (20.0-28.8) | 1072 | 25.8 (22.2-29.8) | 2,314 |  |
| **Zanzibar** |  |  |  |  |  |  |  |
| Private for-profit outlet |  |  |  |  |  |  |  |
| *Health facility/pharmacy* | 73.2 | 82 | 56.3 | 16 | 70.4 | 98 |  |
| *Drug store* | 56.8 | 88 | 45.8 | 24 | 54.5 | 112 |  |
| *General retailer/itinerant* | 66.7 | 3 | 66.7 | 3 | 66.7 | 6 |  |
| *Total* | 64.7 | 173 | 51.2 | 43 | 62.0 | 216 |  |
| CI = Confidence interval; No confidence intervals are shown for Zanzibar as a full census was carried out. | | | | | | | |
