# Supplementary material for: Communicating the AMFm message: exploring the effect of communication and training interventions on private for-profit provider awareness and knowledge related to a multi-country anti-malarial subsidy intervention
Source: Malar J. 2014 Feb 4;13:46. doi: 10.1186/1475-2875-13-46 (PMC3924415; doi:10.1186/1475-2875-13-46)
Supplement: Additional file 4 — Providers stating the correct recommended retail price (RRP) for anti-malarials with the AMFm logo at endline (2011). Providers stating the correct recommended retail price (RRP) for anti-malarials with the AMFm logo at endline (2011) (i.e. Providers stating the correct RRP for anti-malarials with the AMFm logo (n) as a percentage of outlets with anti-malarials in stock at the time of the survey visit (N)) at endline (2011), by anti-malarial outlet type category and urban and rural location.Note: No data are shown for Madagascar as an RRP was not set for co-paid ACTs in this country, CI = Confidence interval; No confidence intervals are shown for Zanzibar as a full census was carried out. [file 1475-2875-13-46-S4.docx]

| **Table web 3: Providers stating the correct recommended retail price (RRP) for antimalarials with the AMFm logo at endline (2011)** | | | | | | |
| --- | --- | --- | --- | --- | --- | --- |
| Providers stating the correct RRP for antimalarials with the AMFm logo (n) as a percentage of outlets with antimalarials in stock at the time of the survey visit (N), by urban-rural location and type of outlet, according to country | | | | | | |
|  | Urban | | Rural | | Total | |
| **Country/Type of outlet** | % (95% CI) | N | % (95% CI) | N | % (95% CI) | N |
| **Ghana** |  |  |  |  |  |  |
| Private for-profit outlet |  |  |  |  |  |  |
| *Health facility/pharmacy* | 94.7 (91.1-96.9) | 269 | 85.3 (62.1-95.3) | 26 | 93.5 (89.3-96.1) | 295 |
| *Drug store* | 86.0 (79.6-90.7) | 202 | 59.4 (45.7-71.7) | 140 | 75.6 (67.9-81.9) | 342 |
| *General retailer/itinerant* | 64.5 (20.5-92.8) | 3 | 0 | 3 | 32.1 (9.4-68.5) | 6 |
| *Total* | 88.1 (82.9-91.9) | 474 | 60.2 (47.3-71.8) | 169 | 78.6 (72.0-84.0) | 643 |
| **Kenya** |  |  |  |  |  |  |
| Private for-profit outlet |  |  |  |  |  |  |
| *Health facility/pharmacy* | 74.0 (66.6-80.3) | 407 | 76.6 (69.3-82.6) | 112 | 75.6 (70.4-80.2) | 519 |
| *Drug store* | 90.8 (86.1-94.0) | 329 | 91.5 (82.4-96.1) | 145 | 91.2 (86.0-94.6) | 474 |
| *General retailer/itinerant* | 36.4 (25.9-48.4) | 155 | 40.8 (29.2-53.5) | 223 | 40.0 (30.2-50.6) | 378 |
| *Total* | 73.7 (67.0-79.4) | 891 | 64.9 (54.4-74.1) | 480 | 67.6 (60.1-74.3) | 1371 |
| **Madagascar** |  |  |  |  |  |  |
| Private for-profit outlet |  |  |  |  |  |  |
| *Health facility/pharmacy* |  |  |  |  |  |  |
| *Drug store* |  |  |  |  |  |  |
| *General retailer/itinerant* |  |  |  |  |  |  |
| *Total* |  |  |  |  |  |  |
| **Niger** |  |  |  |  |  |  |
| Private for-profit outlet |  |  |  |  |  |  |
| *Health facility/pharmacy* | 50.0 (40.7-59.2) | 95 | 100 | 4 | 52.8 (43.3-62.1) | 99 |
| *Drug store* | 67.4 (42.1-85.4) | 15 | 30.7 (5.6-77.0) | 3 | 47.0 (22.2-73.4) | 18 |
| *General retailer/itinerant* | 8.7 (6.7-11.1) | 708 | 1.5 (0.8-2.9) | 509 | 3.5 (2.6-4.7) | 1217 |
| *Total* | 11.6 (9.8-13.8) | 818 | 1.8 (1.0-3.3) | 516 | 4.6 (3.6-5.9) | 1334 |
| **Nigeria** |  |  |  |  |  |  |
| Private for-profit outlet |  |  |  |  |  |  |
| *Health facility/pharmacy* | 4.1 (0.5-24.6) | 94 | 3.3 (0.5-19.1) | 31 | 3.8 (0.9-15.0) | 125 |
| *Drug store* | 1.7 (0.8-3.5) | 797 | 1.8 (0.5-5.7) | 360 | 1.7 (0.9-3.3) | 1157 |
| *General retailer/itinerant* | 0 | 72 | 0 | 19 | 0 | 91 |
| *Total* | 1.8 (0.9-3.7) | 963 | 1.8 (0.6-5.1) | 410 | 1.8 (1.0-3.3) | 1373 |
| **Tanzania - mainland** |  |  |  |  |  |  |
| Private for-profit outlet |  |  |  |  |  |  |
| *Health facility/pharmacy* | 70.7 (58.8-80.3) | 313 | 7.4 (2.4-21.0) | 16 | 54.0 (37.0-70.1) | 329 |
| *Drug store* | 50.9 (42.5-59.3) | 256 | 54.2 (45.0-63.2) | 113 | 52.8 (46.4-59.1) | 369 |
| *General retailer/itinerant* | 31.3 (3.7-84.2) | 4 | 34.6 (26.2-44.0) | 12 | 34.3 (25.7-44.2) | 16 |
| *Total* | 53.5 (45.7-61.2) | 573 | 50.3 (41.3-59.2) | 141 | 51.7 (45.5-57.8) | 714 |
| **Uganda** |  |  |  |  |  |  |
| Private for-profit outlet |  |  |  |  |  |  |
| *Health facility/pharmacy* | 0.9 (0.5-1.6) | 809 | 1.3 (0.3-4.9) | 387 | 1.1 (0.5-2.6) | 1196 |
| *Drug store* | 0.1 (0.0-0.9) | 433 | 0.4 (0.1-1.1) | 675 | 0.3 (0.1-1.0) | 1108 |
| *General retailer/itinerant* | 0 | 4 | 0 | 14 | 0 | 18 |
| *Total* | 0.6 (0.3-1.1) | 1246 | 0.6 (0.2-1.4) | 1076 | 0.6 (0.3-1.1) | 2322 |
| **Zanzibar** |  |  |  |  |  |  |
| Private for-profit outlet |  |  |  |  |  |  |
| *Health facility/pharmacy* | 91.5 ( | 82 | 75.0 | 16 | 88.8 | 98 |
| *Drug store* | 76.1 | 88 | 66.7 | 24 | 74.1 | 112 |
| *General retailer/itinerant* | 66.7 | 3 | 66.7 | 3 | 66.7 | 6 |
| *Total* | 83.2 | 173 | 69.8 | 43 | 80.6 | 216 |
| Note: No data are shown for Madagascar as an RRP was not set for co-paid ACTs in this country, CI = Confidence interval; No confidence intervals are shown for Zanzibar as a full census was carried out. | | | | | | |
