# Supplementary material for: Communicating the AMFm message: exploring the effect of communication and training interventions on private for-profit provider awareness and knowledge related to a multi-country anti-malarial subsidy intervention
Source: Malar J. 2014 Feb 4;13:46. doi: 10.1186/1475-2875-13-46 (PMC3924415; doi:10.1186/1475-2875-13-46)
Supplement: Additional file 5 — Provider knowledge of first-line anti-malarial treatment at baseline (2010) and endline (2011). Provider knowledge of first-line anti-malarial treatment at baseline (2010) and endline (2011) (i.e. Percentage of providers able to correctly identify the anti-malarial for first-line treatment (n) among outlets with anti-malarials in stock at the time of the survey visit (N)) baseline (2010) and at endline (2011), by anti-malarial outlet type category and urban and rural location.Note: Nigeria baseline data collection was conducted in 2009. CI = Confidence interval; No confidence intervals are shown for Zanzibar as a full census was carried out. [file 1475-2875-13-46-S5.docx]

| Table web 4: Provider knowledge of first line antimalarial treatment at baseline (2010) and endline (2011) | | | | | | | | | | | | |
| --- | --- | --- | --- | --- | --- | --- | --- | --- | --- | --- | --- | --- |
| Percentage of providers able to correctly identify the antimalarial for first line treatment (n) among outlets with antimalarials in stock at the time of the survey visit (N), by urban-rural location and type of outlet, according to country | | | | | | | | | | | | |
| **Country/Type of outlet** | **BASELINE** | | | | | | **ENDLINE** | | | | | |
|  | Urban | | Rural | | Total | | Urban | | Rural | | Total | |
|  | % (95% CI) | N | % (95% CI) | N | % (95% CI) | N | % (95% CI) | N | % (95% CI) | N | % (95% CI) | N |
| **Ghana** |  |  |  |  |  |  |  |  |  |  |  |  |
| Private for-profit outlet |  |  |  |  |  |  |  |  |  |  |  |  |
| *Health facility/pharmacy* | 88.5 (82.1-92.8) | 314 | 88.9 (75.2-95.5) | 62 | 88.6 (82.7-92.7) | 376 | 97.5 (93.5-99.1) | 270 | 100.0 | 26 | 97.9 (94.4-99.2) | 296 |
| *Drug store* | 78.6 (73.1-83.1) | 210 | 69.7 (63.7-75.1) | 331 | 70.8 (65.5-75.6) | 541 | 86.2 (76.1-92.5) | 202 | 70.8 (59.8-79.8) | 140 | 80.2 (72.8-86.0) | 342 |
| *General retailer/itinerant* | 44.3 (9.3-86.0) | 2 | 32.1 (11.7-62.8) | 3 | 33.6 (14.3-60.5) | 5 | 64.5 (20.5-92.8) | 3 | 32.7 (3.8-85.7) | 3 | 48.5 (16.0-82.4) | 6 |
| *Total* | 83.0 (78.7-86.6) | 526 | 70.7 (65.0-75.8) | 396 | 73.2 (68.5-77.4) | 922 | 89.0 (81.2-93.9) | 475 | 72.3 (61.4-81.1) | 169 | 83.3 (76.9-88.2) | 644 |
| **Kenya** |  |  |  |  |  |  |  |  |  |  |  |  |
| Private for-profit outlet |  |  |  |  |  |  |  |  |  |  |  |  |
| *Health facility/pharmacy* | 88.3 (80.4-93.3) | 363 | 80.7 (67.9-89.2) | 103 | 84.2 (75.6-90.1) | 466 | 91.8 (87.1-94.9) | 408 | 83.7 (75.5-89.6) | 113 | 86.8 (81.5-90.8) | 521 |
| *Drug store* | 82.9 (77.9-87.0) | 269 | 54.5 (40.1-68.1) | 156 | 60.7 (45.4-74.2) | 425 | 88.0 (85.2-90.4) | 328 | 86.2 (79.9-90.7) | 145 | 86.9 (83.2-90) | 473 |
| *General retailer/itinerant* | 16.6 (11.1-24.1) | 239 | 15.5 (9.6-24.1) | 323 | 15.7 (10.6-22.7) | 562 | 26.0 (14.2-42.9) | 156 | 33.9 (21.0-49.7) | 220 | 32.5 (21.6-45.7) | 376 |
| *Total* | 62.8 (54.9-70.1) | 871 | 39.0 (32.7-45.8) | 582 | 44.9 (39.1-50.9) | 1453 | 75.5 (67.5-82.0) | 892 | 61.8 (50.5-72.0) | 478 | 66.1 (57.9-73.4) | 1370 |
| **Madagascar** |  |  |  |  |  |  |  |  |  |  |  |  |
| Private for-profit outlet |  |  |  |  |  |  |  |  |  |  |  |  |
| *Health facility/pharmacy* | 68.1 (62-73.7) | 122 | 89.6 (51.2-98.6) | 9 | 77.5 (64.7-86.6) | 131 | 77.0 (69.8-82.9) | 105 | 66.5 (27.6-91.2) | 12 | 72.2 (52.9-85.8) | 117 |
| *Drug store* | 58.1 (51-64.8) | 28 | 37.6 (28.3-47.9) | 227 | 45.4 (36.6-54.5) | 255 | 84.4 (76.5-90.0) | 28 | 51.0 (43.8-58.2) | 346 | 54.5 (47.5-61.4) | 374 |
| *General retailer/itinerant* | 3.3 (2.4-4.5) | 1209 | 7.7 (3.9-14.6) | 244 | 6.8 (3.7-12.3) | 1453 | 13.1 (9.5-17.8) | 740 | 13.7 (9.4-19.6) | 403 | 13.7 (9.8-18.7) | 1143 |
| *Total* | 16.9 (10.5-26.1) | 1359 | 11.3 (7.0-17.6) | 480 | 12.5 (8.7-17.7) | 1839 | 26.7 (22.6-31.2) | 873 | 18.1 (13.9-23.1) | 761 | 19.4 (15.7-23.6) | 1634 |
| **Niger** |  |  |  |  |  |  |  |  |  |  |  |  |
| Private for-profit outlet |  |  |  |  |  |  |  |  |  |  |  |  |
| *Health facility/pharmacy* | 76.5 (66.1-84.5) | 106 | 96.4 (70.1-99.7) | 12 | 79.4 (69.4-86.8) | 118 | 82.2 (73.9-88.3) | 95 | 100.0 | 4 | 83.2 (75.2-89.0) | 99 |
| *Drug store* | 59.3 (17.4-90.9) | 14 | 61.5 (22.0-90.0) | 7 | 60.7 (29.4-85.1) | 21 | 78.5 (58.4-90.5) | 15 | 100.0 | 3 | 90.5 (77.6-96.3) | 18 |
| *General retailer/itinerant* | 15.5 (8.5-26.4) | 617 | 8.3 (5.7-11.9) | 792 | 9.7 (7.0-13.3) | 1409 | 34.4 (27.7-41.9) | 710 | 22.5 (17.7-28.1) | 510 | 25.7 (21.7-30.2) | 1220 |
| *Total* | 19.2 (12.5-28.4) | 737 | 8.9 (6.3-12.6) | 811 | 11.1 (8.4-14.5) | 1548 | 37.6 (31.3-44.3) | 820 | 23.0 (18.2-28.7) | 517 | 27.2 (23.2-31.6) | 1337 |
| **Nigeria** |  |  |  |  |  |  |  |  |  |  |  |  |
| Private for-profit outlet |  |  |  |  |  |  |  |  |  |  |  |  |
| *Health facility/pharmacy* | 32.8 (26.4-40.0) | 711 | 23.6 (18.4-29.8) | 24 | 25.8 (20.9-31.4) | 735 | 77.9 (70.2-84.2) | 99 | 47.6 (32.3-63.3) | 31 | 66.9 (57.9-74.8) | 130 |
| *Drug store* | 15.0 (10.4-21.1) | 693 | 9.4 (5.2-16.5) | 254 | 14.1 (10.1-19.4) | 947 | 51.4 (39.9-62.8) | 804 | 50.7 (40.0-61.5) | 362 | 51.2 (42.9-59.4) | 1166 |
| *General retailer/itinerant* | 13.7 (6.6-26.4) | 83 | 0 | 16 | 10.9 (5.4-20.6) | 99 | 25.9 (11.8-47.8) | 74 | 23.6 (7.7-53.3) | 19 | 25.3 (13.4-42.8) | 93 |
| *Total* | 15.0 (11.0-20.3) | 1487 | 10.6 (7.2-15.5) | 294 | 14.3 (10.8-18.6) | 1781 | 52.3 (42.0-62.3) | 977 | 49.4 (39.5-59.3) | 412 | 51.2 (43.9-58.4) | 1389 |
| **Tanzania - mainland** |  |  |  |  |  |  |  |  |  |  |  |  |
| Private for-profit outlet |  |  |  |  |  |  |  |  |  |  |  |  |
| *Health facility/pharmacy* | 96.5 (93.1-98.3) | 220 | 100.0 | 12 | 97.2 (94.3-98.6) | 232 | 97.3 (89.3-99.4) | 321 | 99.5 (95.3-100) | 16 | 97.9 (91.7-99.5) | 337 |
| *Drug store* | 94.1 (86.5-97.5) | 88 | 90.8 (85.2-94.4) | 149 | 91.9 (87.7-94.8) | 237 | 96.0 (92.5-97.9) | 259 | 95.9 (87.0-98.8) | 113 | 95.9 (91.6-98.1) | 372 |
| *General retailer/itinerant* | 0 | 1 | 70.7 (58.5-80.4) | 70 | 70.2 (58.2-80.0) | 71 | 81.9 (30.9-97.9) | 5 | 91.0 (82.9-95.4) | 12 | 90.2 (82.9-94.5) | 17 |
| *Total* | 93.9 (88.2-96.9) | 309 | 82.7 (75.1-88.4) | 231 | 85.6 (79.2-90.3) | 540 | 96.0 (92.5-98.0) | 585 | 95.5 (88.4-98.3) | 141 | 95.7 (92.1-97.7) | 726 |
| **Uganda** |  |  |  |  |  |  |  |  |  |  |  |  |
| Private for-profit outlet |  |  |  |  |  |  |  |  |  |  |  |  |
| *Health facility/pharmacy* | 72.3 (67.0-77.1) | 389 | 68.7 (61.1-75.4) | 356 | 70.5 (65.4-75.1) | 745 | 83.1 (81.0-85.0) | 814 | 79.3 (72.5-84.8) | 388 | 81.0 (77.0-84.5) | 1202 |
| *Drug store* | 78.5 (55.3-91.5) | 72 | 75.6 (69.4-80.8) | 745 | 76.0 (70-81.1) | 817 | 76.9 (72.1-81.0) | 435 | 73.0 (67.8-77.6) | 676 | 73.5 (69.1-77.6) | 1111 |
| *General retailer/itinerant* | 59.8 (7.5-96.5) | 2 | 55.3 (36.9-72.4) | 19 | 55.9 (37.3-73) | 21 | 51.5 (11.1-90.1) | 4 | 27.1 (6.6-66.1) | 14 | 28.5 (7.7-65.7) | 18 |
| *Total* | 74.7 (61.1-84.8) | 463 | 73.8 (68.2-78.8) | 1120 | 74.0 (68.8-78.6) | 1583 | 80.3 (78.0-82.3) | 1253 | 73.1 (67.4-78.1) | 1078 | 74.8 (70.4-78.8) | 2,331 |
| **Zanzibar** |  |  |  |  |  |  |  |  |  |  |  |  |
| Private for-profit outlet |  |  |  |  |  |  |  |  |  |  |  |  |
| *Health facility/pharmacy* | 82.2 | 73 | 81.8 | 11 | 82.1 | 84 | 97.6 | 82 | 100.0 | 16 | 98.0 | 98 |
| *Drug store* | 71.9 | 57 | 80.0 | 25 | 74.4 | 82 | 88.6 | 88 | 87.5 | 24 | 88.4 | 112 |
| *General retailer/itinerant* | 100.0 | 1 | 25.0 | 4 | 40.0 | 5 | 66.7 | 3 | 66.7 | 3 | 66.7 | 6 |
| *Total* | 77.9 | 131 | 75.0 | 40 | 77.2 | 171 | 92.5 | 173 | 90.7 | 43 | 92.1 | 216 |
| Note: Nigeria baseline data collection was conducted in 2009. CI = Confidence interval; No confidence intervals are shown for Zanzibar as a full census was carried out. | | | | | | | | | | | | |
