# Supplementary material for: Communicating the AMFm message: exploring the effect of communication and training interventions on private for-profit provider awareness and knowledge related to a multi-country anti-malarial subsidy intervention
Source: Malar J. 2014 Feb 4;13:46. doi: 10.1186/1475-2875-13-46 (PMC3924415; doi:10.1186/1475-2875-13-46)
Supplement: Additional file 6 — Provider knowledge of dosing regimen for quality-assured ACT (QAACT) for a child, at baseline (2010) and endline (2011). Provider knowledge of dosing regimen for quality-assured ACT (QAACT) for a child, at baseline (2010) and endline (2011) (i.e. Percentage of providers able to describe correctly the dosing regimen for quality-assured ACT for a child under 2 years of age (<10 kg) (n) among outlets with QAACT in stock at the time of the survey visit (N)) baseline (2010) and at endline (2011), by anti-malarial outlet type category and urban and rural location. Note: “describe correctly” implies that the respondent correctly stated the number of tablets that should be taken at a time, the number of times the medicine should be taken per day and the duration of the dose in number of days for child under 2 years (10kg) for a specific product which they selected from the quality-assured ACTs that they stocked. These data are not available for Madagascar and Nigeria at baseline, as they were not collected in the ACTwatch survey. Nigeria baseline data collection was conducted in 2009. CI = Confidence interval; No confidence intervals are shown for Zanzibar as a full census was carried out. [file 1475-2875-13-46-S6.docx]

| **Table web 5: Provider knowledge of dosing regimen for quality-assured ACTs (QAACTs) for a child, at baseline (2010) and endline (2011)** | | | | | | | | | | | | |
| --- | --- | --- | --- | --- | --- | --- | --- | --- | --- | --- | --- | --- |
| Percentage of providers able to describe correctly the dosing regimen for quality-assured ACTs for a child under 2 years of age (<10kg) (n) among outlets with QAACTs in stock at the time of the survey visit (N), by urban-rural location and type of outlet, according to country | | | | | | | | | | | | |
| **Country/Type of outlet** | **BASELINE** | | | | | | **ENDLINE** | | | | | |
|  | Urban | | Rural | | Total | | Urban | | Rural | | Total | |
|  | % (95% CI) | N | % (95% CI) | N | % (95% CI) | N | % (95% CI) | N | % (95% CI) | N | % (95% CI) | N |
| **Ghana** |  |  |  |  |  |  |  |  |  |  |  |  |
| Private for-profit outlet |  |  |  |  |  |  |  |  |  |  |  |  |
| *Health facility/pharmacy* | 45.6 (39.5-51.9) | 241 | 47.1 (31.5-63.3) | 29 | 46.0 (39.9-52.2) | 270 | 70.3 (63.5-76.2) | 238 | 63.9 (39.8-82.5) | 21 | 69.4 (62.8-75.3) | 259 |
| *Drug store* | 8.6 (4.9-14.9) | 61 | 27.2 (18.4-38.2) | 57 | 23.7 (16.3-33.1) | 118 | 40.3 (34.4-46.4) | 151 | 43.9 (35.9-52.2) | 96 | 41.6 (36.9-46.6) | 247 |
| *General retailer/itinerant* | - | 0 | - | 0 | - | 0 | 35.5 (7.2-79.5) | 3 | 0 | 2 | 21.3 (4.3-61.7) | 5 |
| *Total* | 33.7 (27.9-40.1) | 302 | 29.9 (21.7-39.5) | 86 | 31.4 (25.8-37.6) | 388 | 49.1 (42.7-55.5) | 392 | 44.9 (37.9-52.2) | 119 | 47.8 (42.8-52.7) | 511 |
| **Kenya** |  |  |  |  |  |  |  |  |  |  |  |  |
| Private for-profit outlet |  |  |  |  |  |  |  |  |  |  |  |  |
| *Health facility/pharmacy* | 80.2 (63.4-90.5) | 229 | 68.2 (47.3-83.7) | 43 | 75.2 (62.0-84.9) | 272 | 61.6 (49.0-72.9) | 345 | 65.0 (56.6-72.5) | 88 | 63.7 (56.5-70.2) | 433 |
| *Drug store* | 66.2 (48.7-80.2) | 140 | 56.3 (43.7-68.1) | 30 | 60.4 (50.3-69.7) | 170 | 70.0 (62.2-76.7) | 305 | 60.3 (49.1-70.5) | 120 | 64.4 (56.7-71.3) | 425 |
| *General retailer/itinerant* | - | 0 | - | 0 | - | 0 | 40.4 (19.4-65.5) | 30 | 41.7 (28.3-56.5) | 67 | 41.6 (29.2-55.0) | 97 |
| *Total* | 74.1 (57.2-85.9) | 369 | 61.0 (51.8-69.4) | 73 | 67.4 (57.9-75.7) | 442 | 65.8 (57.9-72.9) | 680 | 57.7 (51.1-64.0) | 275 | 60.6 (55.4-65.7) | 955 |
| **Madagascar** |  |  |  |  |  |  |  |  |  |  |  |  |
| Private for-profit outlet | - | - | - | - | - | - |  |  |  |  |  |  |
| *Health facility/pharmacy* | - | - | - | - | - | - | 51.5 (40.7-62.2) | 87 | 100.0 | 2 | 56.0 (42.8-68.3) | 89 |
| *Drug store* | - | - | - | - | - | - | 39.7 (20.7-62.5) | 23 | 42.9 (35.9-50.2) | 173 | 42.4 (35.5-49.6) | 196 |
| *General retailer/itinerant* | - | - | - | - | - | - | 0 | 2 | 0 | 4 | 0 | 6 |
| *Total* | - | - | - | - | - | - | 48.0 (38.4-57.8) | 112 | 37.7 (30.4-45.6) | 179 | 41.6 (35.6-47.7) | 291 |
| **Niger** |  |  |  |  |  |  |  |  |  |  |  |  |
| Private for-profit outlet |  |  |  |  |  |  |  |  |  |  |  |  |
| *Health facility/pharmacy* | 47.7 (29.2-66.8) | 65 | 94.6 (52.5-99.6) | 3 | 56.3 (34.3-76.1) | 68 | 54.1 (37.8-69.6) | 65 | 91.6 (61.9-98.6) | 4 | 57.9 (41.7-72.5) | 69 |
| *Drug store* | 97.1 (78.4-99.7) | 8 | 100.0 | 1 | 98.0 (84.5-99.8) | 9 | 88.4 (69.8-96.2) | 9 | 48.7 (8.5-90.7) | 2 | 67.1 (29.7-90.8) | 11 |
| *General retailer/itinerant* | 58.9 (43.5-72.6) | 49 | 65.4 (46.3-80.6) | 29 | 62.9 (50.3-74.0) | 78 | 45.8 (37.4-54.4) | 137 | 36.8 (23.1-52.9) | 48 | 41.0 (32.3-50.3) | 185 |
| *Total* | 59.9 (48.5-70.2) | 122 | 67.6 (48.7-82.1) | 33 | 64.1 (53.1-73.9) | 155 | 48.3 (40.9-55.7) | 211 | 38.0 (24.8-53.3) | 54 | 43.1 (35.1-51.4) | 265 |
| **Nigeria** |  |  |  |  |  |  |  |  |  |  |  |  |
| Private for-profit outlet | - | - | - | - | - | - |  |  |  |  |  |  |
| *Health facility/pharmacy* | - | - | - | - | - | - | 55.6 (31.5-77.2) | 64 | 65.8 (36.2-86.7) | 13 | 57.4 (37.0-75.6) | 77 |
| *Drug store* | - | - | - | - | - | - | 48.3 (37.6-59.1) | 470 | 61.0 (49.7-71.1) | 197 | 53.3 (44.5-61.9) | 667 |
| *General retailer/itinerant* | - | - | - | - | - | - | 49.7 (23.2-76.4) | 20 | 100.0 | 3 | 54.4 (28.6-78.1) | 23 |
| *Total* | - | - | - | - | - | - | 49.3 (38.7-60.0) | 554 | 61.5 (50.7-71.2) | 213 | 53.7 (45.1-62.1) | 767 |
| **Tanzania - mainland** |  |  |  |  |  |  |  |  |  |  |  |  |
| Private for-profit outlet |  |  |  |  |  |  |  |  |  |  |  |  |
| *Health facility/pharmacy* | 81.2 (71.6-88.1) | 95 | 8.3 (0.7-54.9) | 3 | 72.7 (55-85.3) | 98 | 88.0 (79.1-93.4) | 278 | 96.1 (83.0-99.2) | 12 | 89.9 (81.8-94.6) | 290 |
| *Drug store* | 43.8 (14.7-77.9) | 9 | 70.4 (34.8-91.4) | 12 | 59.4 (36-79.2) | 21 | 87.9 (75.6-94.4) | 153 | 90.8 (77.8-96.6) | 67 | 89.5 (81.2-94.4) | 220 |
| *General retailer/itinerant* | - | 0 | 35.7 (4.4-87.1) | 3 | 35.7 (4.4-87.1) | 3 | 63.1 (12.0-95.5) | 3 | 100.0 | 1 | 81.6 (28.5-98.0) | 4 |
| *Total* | 61.1 (43.7-76.1) | 104 | 59.0 (30.6-82.4) | 18 | 60.0 (42.6-75.2) | 122 | 87.5 (76.6-93.8) | 434 | 91.2 (78.5-96.7) | 80 | 89.5 (81.7-94.2) | 514 |
| **Uganda** |  |  |  |  |  |  |  |  |  |  |  |  |
| Private for-profit outlet |  |  |  |  |  |  |  |  |  |  |  |  |
| *Health facility/pharmacy* | 63.3 (41.5-80.7) | 197 | 75.7 (58.7-87.2) | 75 | 67.8 (52.9-79.7) | 272 | 75.2 (70.5-79.5) | 687 | 81.4 (72.1-88.2) | 299 | 78.6 (72.7-83.5) | 986 |
| *Drug store* | 23.8 (3.6-72.2) | 6 | 67.4 (57.3-76.1) | 65 | 62.5 (49.8-73.6) | 71 | 81.3 (74.6-86.6) | 296 | 79.7 (74.3-84.2) | 407 | 80.0 (75.5-83.8) | 703 |
| *General retailer/itinerant* | - | 0 | 0 | 1 | 0 | 1 | 37.6 (5.0-87.3) | 3 | 40.7 (26.4-56.8) | 9 | 40.5 (26.5-56.3) | 12 |
| *Total* | 54.9 (27.0-80.0) | 203 | 68.4 (60.4-75.4) | 141 | 64.1 (52.9-73.9) | 344 | 77.4 (73.2-81.1) | 986 | 78.9 (73.4-83.5) | 715 | 78.5 (74.3-82.1) | 1701 |
| **Zanzibar** |  |  |  |  |  |  |  |  |  |  |  |  |
| Private for-profit outlet |  |  |  |  |  |  |  |  |  |  |  |  |
| *Health facility/pharmacy* | 20.0 | 10 | 0 | 1 | 18.2 | 11 | 53.7 | 67 | 50.0 | 12 | 53.2 | 79 |
| *Drug store* | 0 | 2 | - | 0 | 0 | 2 | 46.4 | 56 | 43.8 | 16 | 45.8 | 72 |
| *General retailer/itinerant* | - | 0 | - | 0 | - | 0 | 0 | 1 | 0 | 2 | 0 | 3 |
| *Total* | 16.7 | 12 | 0 | 1 | 15.4 | 13 | 50 | 124 | 43.3 | 30 | 48.7 | 154 |
| Note: “describe correctly” implies that the respondent correctly stated the number of tablets that should be taken at a time, the number of times the medicine should be taken per day and the duration of the dose in number of days for child under 2 years (10kg) for a specific product which they selected from the quality-assured ACTs that they stocked. These data are not available for Madagascar and Nigeria at baseline, as they were not collected in the ACTwatch survey. Nigeria baseline data collection was conducted in 2009. CI = Confidence interval; No confidence intervals are shown for Zanzibar as a full census was carried out. | | | | | | | | | | | | |
